# Supplementary material for: The impact of climate suitability, urbanisation, and connectivity on the expansion of dengue in 21st century Brazil
Source: PLoS Negl Trop Dis. 2021 Dec 9;15(12):e0009773. doi: 10.1371/journal.pntd.0009773 (PMC8691609; doi:10.1371/journal.pntd.0009773)
Supplement: S1 Text — Additional information about the methods and materials used in this study and results of sensitivity analyses. (DOCX) [file pntd.0009773.s001.docx]

S1 Document: Supplementary material

# Methods and materials

## Dengue surveillance and outbreak definitions in Brazil

Monthly dengue case data are freely available from Brazil's Notifiable Diseases Information System (SINAN), via the Health Information Department, DATASUS (https://datasus.saude.gov.br/informacoes-de-saude-tabnet/). Although notification of a suspected dengue case is mandatory in Brazil, the surveillance system is predominantly passive, which means that many mild and asymptomatic cases may be missed. One investigation of the Brazilian dengue surveillance system estimated that there were 12 actual infections per reported case overall, which rose to over 17 in periods of high incidence [1]. Rather than use dengue case data which differs in accuracy between regions, and between epidemic and non-epidemic periods, we aggregated the cases by year and converted them into a binary outbreak indicator where cases exceeded some outbreak threshold. Several methods have been used to define outbreak thresholds Brazil, including a monthly moving average, where historical data are used to estimate the expected number of cases within a region [2,3], and a fixed threshold based on the dengue incidence rate (DIR), defined by the Brazilian Ministry of Health as the number of cases per 100,000 residents [4]. We chose to use a fixed threshold approach as the mean incidence was heavily influenced by outbreak years, making the probability of detecting an outbreak inconsistent between municipalities. Our primary analysis used an outbreak threshold of more than 300 cases per 100,000 residents, defined as 'high risk' by the Brazilian Ministry of Health. We also tested a 'medium risk' indicator, defined as more than 100 cases per 100,000 residents [4]. The annual DIR was calculated using estimates of the annual population for each municipality obtained from the Brazilian Institute of Statistics and Geography (IBGE) via DATASUS (https://datasus.saude.gov.br/populacao-residente). As an alternative, we used the 75th percentile of the DIR per municipality with a minimum threshold value equivalent to 5 cases per year to avoid very low cases triggering an outbreak in 'protected' areas. The 75th percentile of the DIR was calculated using all available data from 2001 - 2020 for each municipality. Many municipalities in previously 'protected' areas such as South Brazil and the western Amazon had lower thresholds using this method than the fixed thresholds used by the Brazilian Ministry of Health (Fig A). However, the threshold was much higher (up to a maximum of DIR = 3275) in regions which had experienced high levels of dengue transmission in the past.


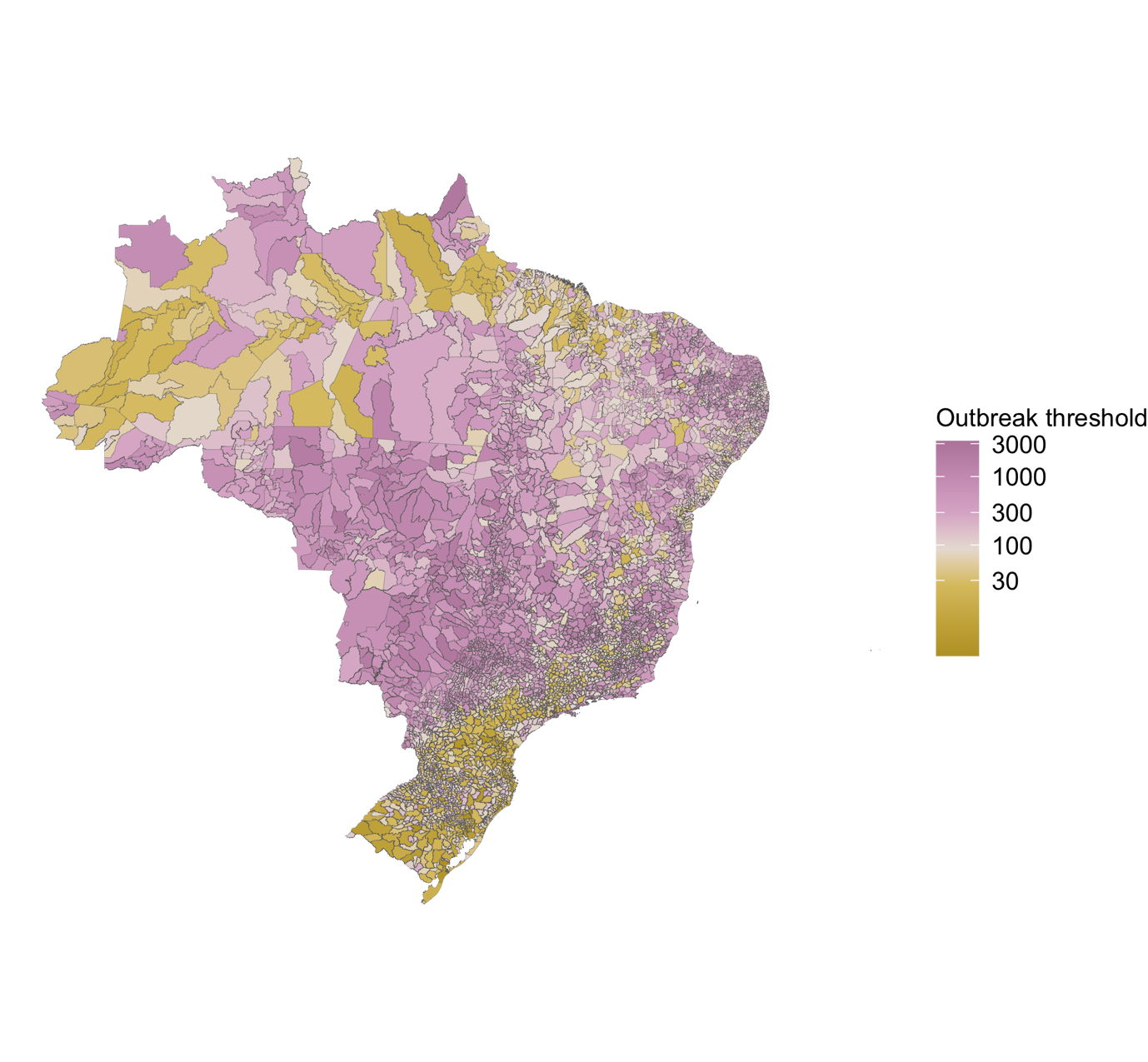


**Fig A: The outbreak threshold for each municipality based on the 75th percentile of dengue incidence rates between 2001 - 2020.** Regions with historically low dengue transmission, such as South Brazil, had thresholds below 100 (shown in gold), whilst areas with sustained high transmission such as the Centre-West had much higher thresholds, up to a maximum of 3275. Maps were produced in R using the geobr package [5,6] (hsttps://ipeagit.github.io/geobr/).

## Hydrometeorological factors

In addition to temperature suitability, hydrometeorological conditions such as precipitation and drought have been linked to dengue transmission. Prior studies have found that the risk of dengue increases immediately following extremely wet conditions [7,8], however the level of precipitation considered extreme varies greatly across Brazil between climate systems. To measure the relative wetness of municipalities, we used the self-calibrating Palmer Drought Severity Index (scPDSI). The scPDSI was obtained from the Climate Research Unit gridded Time Series (v4.05) [9,10] for the period January 2001 - December 2020, at a spatial resolution of 0.5° x 0.5°. The PDSI is a widely used measure of meteorological drought ranging from -10 (dry) to 10 (wet) compared to 'normal conditions', with values below -4 and above 4 considered extreme [11,12]. The scPDSI calibrates this index to the 'normal conditions' for each location of interest separately, providing a more spatially comparable measure [10,13]. The scPDSI was aggregated to each municipality using the exactextractr package [14] in R (version 4.0.3) by calculating the mean of the grid boxes lying within each municipality. Grid boxes partially covered by a municipality were weighted by the percentage of area that lay within the municipality.

Most states, particularly those in North Brazil, have experienced increasingly severe drought conditions in recent years. However, there have been several extremely wet events, particularly in the Southeast of the country (Fig B). To understand the relationship between wet conditions and dengue outbreaks, we calculated the number of months per year each municipality took an scPDSI value of 4 and above, considered 'extremely wet' by the scPDSI [10,12]. On average, the number of months considered extremely wet has increased in parts of South Brazil and in Pará, North Brazil, and has reduced in the Amazon and Southeast Brazil (Fig C).


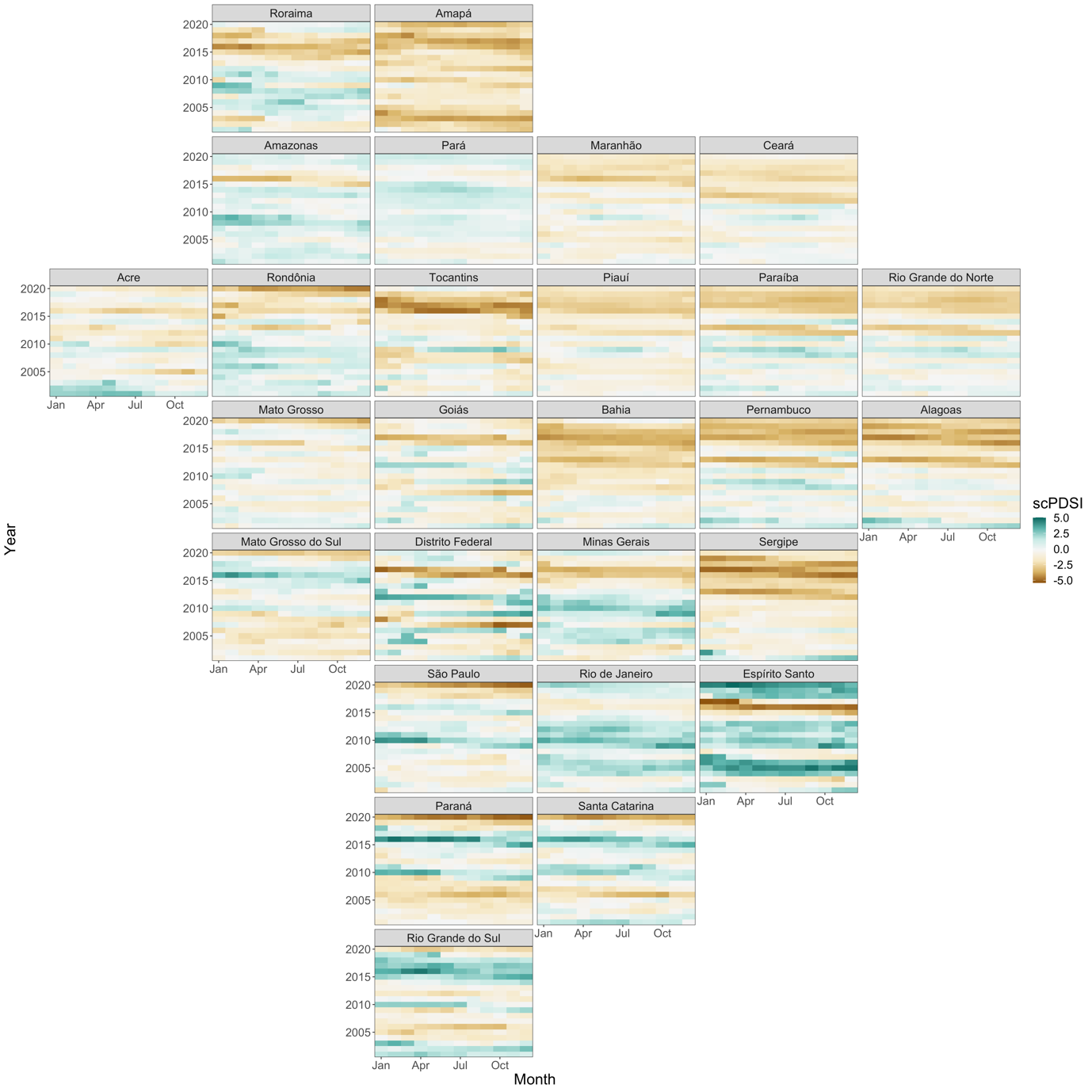


**Fig B: The average monthly self-calibrated Palmer Drought Severity Index (scPDSI) per state from January 2001 - December 2020.** Values below -4 (shown in brown) are considered extremely dry compared to normal conditions, whereas values above 4 (shown in blue) are considered extremely. wet. The north and east of Brazil has experienced increasingly severe droughts in recently years, in contrast states in the Southeast have experienced a number of extremely wet conditions.


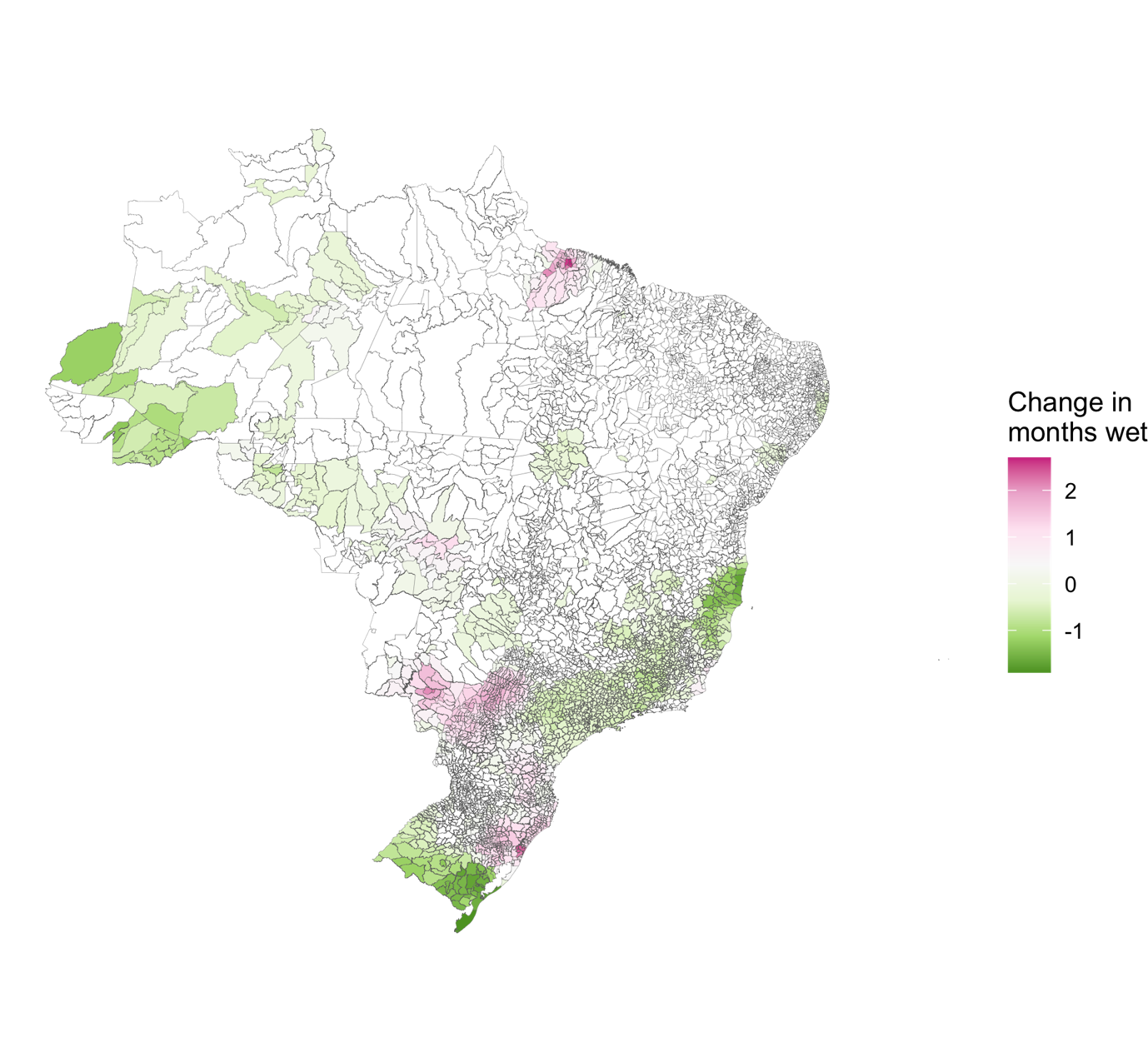


**Fig C: Map showing the difference in the average number of months per year considered extremely wet (scPDSI > 4) between 2001 - 2010 and 2011 - 2020.** The number of months considered extremely wet has increased on average in parts of South and North Brazil (shown in pink). In comparison, the number of extremely wet months per year in the western Amazon and parts of South and Southeast Brazil have reduced. Maps were produced in R using the geobr package [5,6] (https://ipeagit.github.io/geobr/).

## Socioeconomic factors

We obtained information about the percentage of residents in each municipality living in urban areas, the percentage with access to the piped water system, and the percentage that had refuse collected (either privately or using the municipal service) from the 2000 and 2010 censuses via DATASUS. Despite Brazil having the largest economy in South America, it has been the most unequal since 2015 [15]. Access to basic services differs greatly across the country and the traditionally wealthier regions in the South and Southeast have almost universal coverage at the municipality level in contrast to rural parts of the North and Northeast which had little or no access, even in 2010. We found that the level of urbanisation was highly correlated to access to piped water (Fig D, r = 0.656, 95% confidence interval: [0.641, 0.671], p < 0.001) and refuse collection (Fig D, r = 0.794, 95% confidence interval: [0.784, 0.804] p < 0.001) when aggregated to the municipality level. Therefore, access to piped water and refuse collection were not included in the models as they were not useful at explaining the differences within cities at this level of aggregation and would likely introduce multicollinearity into the model.

**
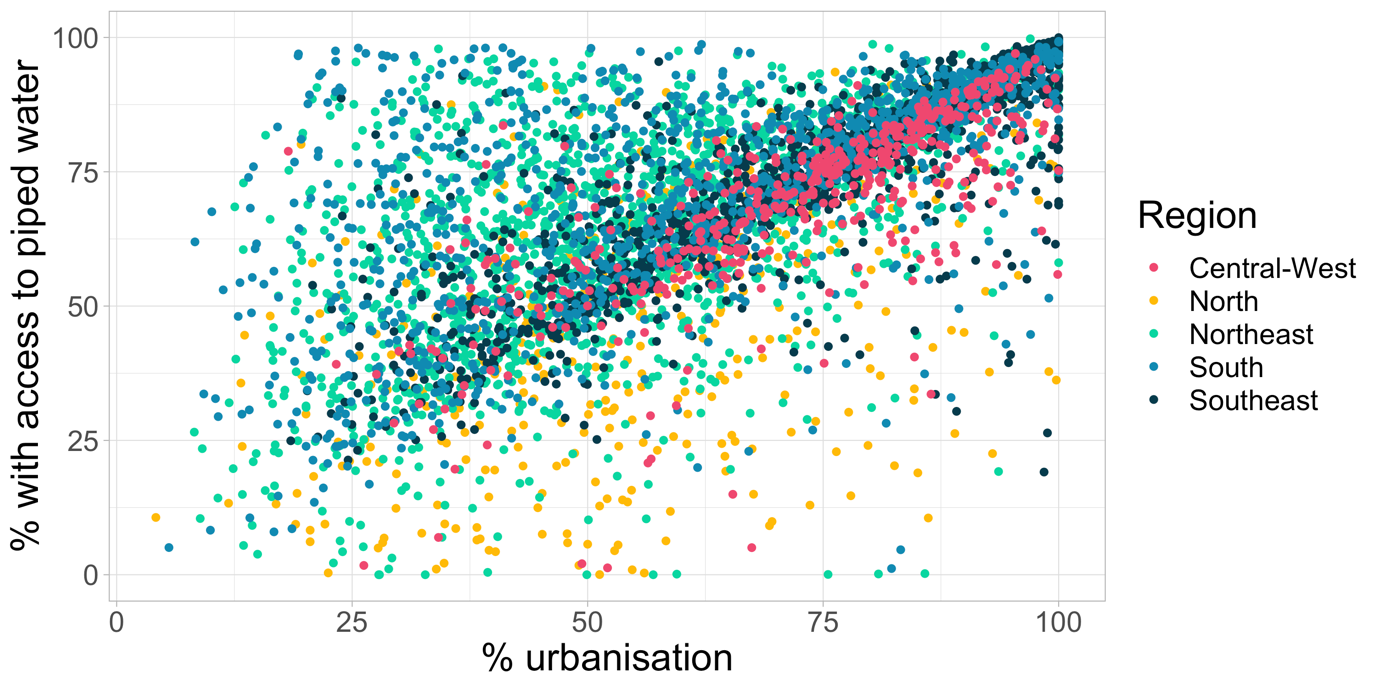
**

**
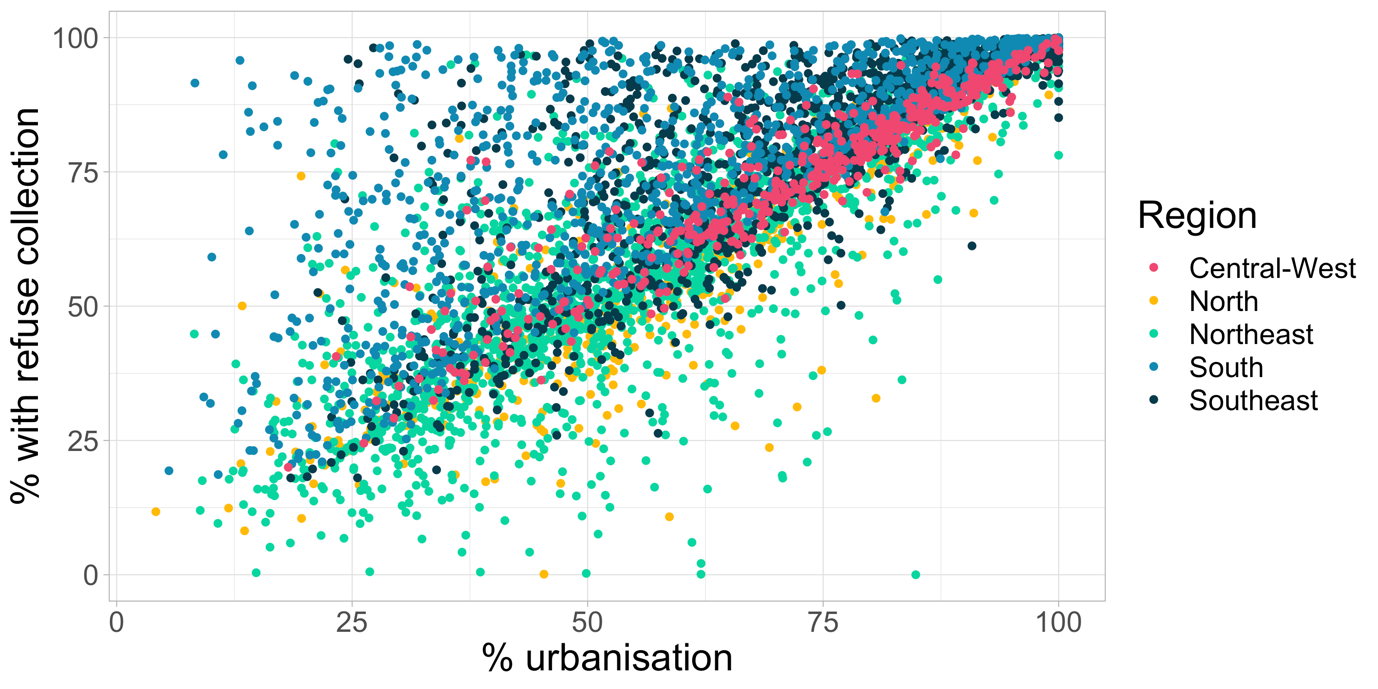
**

**Fig D: Scatterplot comparing the percentage of residents with access to piped water (top) and refuse collection (bottom) to the percentage living in urban areas from the 2010 census.** Access to basic services was highly correlated to the level of urbanisation: highly urban areas had highest access to piped water and refuse collection.

## Hierarchical levels of influence of cities

We extracted the level of influence of cities from the Regions of Influence of Cities (“Regiões de Influência das Cidades”, REGIC) studies carried out by IBGE in 2007 and 2018 [16,17] to use as a proxy for human movement within our models. REGIC aims to recreate the complex urban network of Brazil using information from surveys about the frequency and reasons for the movement of people and goods around the country. The level of influence assigned to each city was based on the number of people travelling to the city but also the number of important institutions that attracts the movement of people from outside the city, such as hospitals, universities, business centres, government agencies, and cultural centres (such as theatres and shopping centres). Cities were classified into five levels:

1. Metropolis: the largest cities in Brazil, with strong connections throughout the entire country. This includes São Paulo, the capital Brasilia, and Rio de Janeiro.
2. Regional capital: large cities which are connected throughout the region in which they are located and to metropoles. This includes state capitals that were not classified as metropoles, such as Rio Branco, Campo Grande and Porto Velho.
3. Sub-regional capital: cities with a lower level of connectivity, mostly connected locally and to the three largest metropoles.
4. Zone centre: smaller cities with influences restricted to their immediate area, often neighbours.
5. Local centre: the smallest cities in the network which typically only serve residents of the municipality and are not connected elsewhere.

There were 12 metropoles, consisting of 203 municipalities, according to the 2007 REGIC study: São Paulo, Rio de Janeiro, Brasilia, Manaus, Belém, Fortaleza, Recife, Salvador, Belo Horizonte, Curitiba, Goiânia and Porto Alegre. In 2018, this increased to 15 metropoles, consisting of 214 municipalities, as Campinas, Florianópolis and Vitória were re-classified from regional capitals to metropoles. The number of regional capitals and sub-regional centres also increased between 2007 and 2018 from 70 to 97 and from 169 to 352 respectively. The number of lower-level cities, zone centre and local centres, both decreased from 556 to 398, and from 4473 to 4037 (S1Table). The distribution of highly connected urban centres is uneven across the country; the South and Southeast regions are particularly well connected, while the North and Northeast contain fewer high-level centres (Fig 3 and S1 Table). The proportion of higher-level centres has increased in each region of Brazil, although the Amazon rainforest remains less connected than other areas (Fig E). Metropoles, regional capitals and sub-regional centres had higher levels of urbanisation, access piped water and refuse collection on average than less connected centres (Fig S6).

**a) b)**


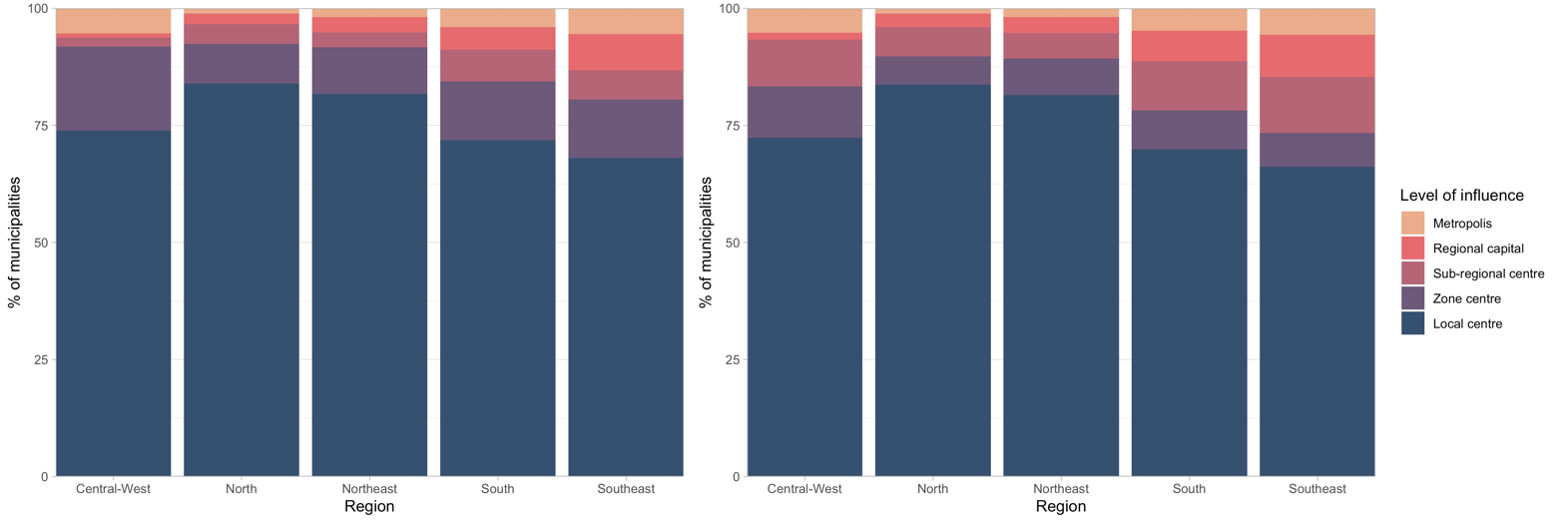


**Fig E: The proportion of cities in each region at each level of influence in the a) 2007 and b) 2018 REGIC study.** The proportion of high-level cities has increased across the country but the North and Northeast still have noticeably less well-connected cities than other regions. The Southeast and South are by far the most connected regions.

## Modelling approach

We formulated a spatio-temporal generalised additive model (GAM) to quantify the relationship between temperature suitability, level of connectivity and socioeconomic conditions on the odds of a municipality experiencing an outbreak. The response variable was a binary outbreak indicator defined as an annual dengue incidence rate of more than 300 cases per 100,000 residents. To account for spatial and temporal patterns in the data, smooth functions of the year and the coordinates of the centroids of municipalities were included in the model. We used thin plate regression splines to represent the smooth (2D) function of the coordinates. Thin plate splines are data-driven and estimate the best fitting function for the data [18]. To account for changes in spatial patterns over the period, we also included a space-time interaction term created by applying a tensor product smooth to the coordinates and the year. Tensor product smooths allow interactions between variables that are measured on different scales (in this case, space and time). The final model equation was as follows:

$$Y_{it}\sim Bernoulli(p_{it})$$

$$logit(p_{it})=\beta_{0}+\sum_{j = 1}^{m} \beta_{j}X_{jit}+ f_{spat}({lon}_{i},{lat}_{i})+f_{time}(t)+f_{int}({lon}_{i},{lat}_{i},t)$$

Where $Y_{it}$, binary outbreak indicator for municipality *i* (*i = 1, ..., 5,560*) in year *t* (*t = 2001, ..., 2020*), is expected to follow a Bernoulli distribution defined by $p_{it}$, the probability of an outbreak. The Bernoulli distribution is a special case of the binomial distribution where the number of trials is equal to 1. $\beta_{j}$ are coefficient estimates associated with covariates $X_{jit}$. $f_{spat}({lon}_{i},{lat}_{i})$ is the spatial smooth field based on the coordinates (${lon}_{i},{lat}_{i}$) of the centroid of municipality *i*, $f_{time}(t)$ is the temporal smooth function applied to year *t* and $f_{int}({lon}_{i},{lat}_{i},t)$ is the spatio-temporal interaction term. This model is a type of structured additive regression (STAR) model which allows for Bayesian interpretations of additive models by specifying prior beliefs on the smooth functions [18,19].

We chose to include the number of months with temperature suitable for dengue transmission, the proportion of residents living in urban areas, the level of influence from the REGIC study, and the prior outbreak indicator as covariates in our final model to address our initial research questions. We also tested the number of months considered extremely wet according to the scPDSI as hydrometeorological factors are also recognised as important drivers of dengue transmission [7,8]. Although this coefficient was statistically significant (adjusted odds ratio: 1.11, 95% credible interval: 1.09, 1.14), the model fit was not improved with the addition of this covariate (S3 Table) and the covariate was excluded from further analysis.

Inference was performed using an empirical Bayesian approach with estimates calculated using restricted maximum likelihood (REML), an approach that has been shown to give more stable estimates than generalised cross validation [20], and more accurate estimates than a full Bayesian approach for binomial models [19]. We used the mgcv package in R [18] to fit the spatio-temporal models and to simulate from the posterior distributions of the coefficients to produce mean estimates and 95% credible intervals.

# Results

## Outbreak threshold comparisons

To check whether our model results were robust to the definition of an outbreak, we compared our primary results to alternative outbreak indicators: over 100 cases per 100,000 residents (considered medium risk by the Brazilian Ministry of Health [21]), and above the 75th percentile of the yearly DIR between 2001 - 2020 for each municipality with a minimum threshold set as over 5 cases per year. Although the models agreed that the odds of an outbreak were significantly increased in highly connected, highly urbanised cities that had previously experienced an outbreak and had a suitable temperature, the coefficient estimates differed (S2 Table). The 75th percentile model had noticeably lower coefficient estimates for each parameter compared to the fixed threshold models (Fig S8). Most credible intervals for the coefficient estimates of the fixed threshold models overlapped, however the odds of experiencing an outbreak in municipalities that had previously was higher in the model using the DIR = 100 threshold (aOR: 2.42, 95% CI: 2.31, 2.56) compared to the DIR = 300 threshold (aOR: 2.03, 95% CI: 1.93, 2.15).

We assessed the model fit of these alternative outbreak threshold models using a receiver operating characteristic (ROC) curve which plots the true positive rate against the true negative rate at different thresholds to test the predictive ability of the model. The area under the ROC curve was calculated as this gives a measure of predictive ability compared to chance, which would return a value of 0.5. The closer the area under the ROC curve is to 1, the better the model fits the data. The predictive ability of models were also compared using the Brier score [22]. The Brier score is the mean squared difference between the observed and expected outcomes; a lower Brier score represents a better fitting model. We found that the fixed threshold models fit the data better according to the ROC curve (Fig S7 and S3 Table), the Brier score also showed that these models had a better predictive ability than the 75th percentile model (S3 Table).

# References

1. Silva MMO, Rodrigues MS, Paploski IAD, Kikuti M, Kasper AM, Cruz JS, et al. Accuracy of Dengue Reporting by National Surveillance System, Brazil. Emerg Infect Dis. 2016;22: 336–339. doi:10.3201/eid2202.150495

2. Badurdeen S, Valladares DB, Farrar J, Gozzer E, Kroeger A, Kuswara N, et al. Sharing experiences: towards an evidence based model of dengue surveillance and outbreak response in Latin America and Asia. BMC Public Health. 2013;13: 607. doi:10.1186/1471-2458-13-607

3. Codeco C, Coelho F, Cruz O, Oliveira S, Castro T, Bastos L. Infodengue: A nowcasting system for the surveillance of arboviruses in Brazil. Rev DÉpidémiologie Santé Publique. 2018;66: S386. doi:10.1016/j.respe.2018.05.408

4. Lowe R, Barcellos C, Coelho CAS, Bailey TC, Coelho GE, Graham R, et al. Dengue outlook for the World Cup in Brazil: An early warning model framework driven by real-time seasonal climate forecasts. Lancet Infect Dis. 2014;14: 619–626. doi:10.1016/S1473-3099(14)70781-9

5. Lee S.A, Economou T., Catão R., Barcellos C., Lowe R. Data and R code to accompany “The impact of climate suitability, urbanisation, and connectivity on the expansion of dengue in 21st century Brazil” (version 1.0.0). 2021. Available: https://github.com/sophie-a-lee/Dengue_expansion

6. Pereira, R.H.M., Gonçalves, C.N. geobr: Loads Shapefiles of Official Spatial Data Sets of Brazil. 2019. Available: https://github.com/ipeaGIT/geobr

7. Lowe R, Lee SA, O’Reilly KM, Brady OJ, Bastos L, Carrasco-Escobar G, et al. Combined effects of hydrometeorological hazards and urbanisation on dengue risk in Brazil: a spatiotemporal modelling study. Lancet Planet Health. 2021;5: e209–e219. doi:10.1016/S2542-5196(20)30292-8

8. Lowe R, Gasparrini A, Meerbeeck CJV, Lippi CA, Mahon R, Trotman AR, et al. Nonlinear and delayed impacts of climate on dengue risk in Barbados: A modelling study. PLOS Med. 2018;15: e1002613. doi:10.1371/journal.pmed.1002613

9. Harris I, Osborn TJ, Jones P, Lister D. Version 4 of the CRU TS monthly high-resolution gridded multivariate climate dataset. Sci Data. 2020;7: 109. doi:10.1038/s41597-020-0453-3

10. van der Schrier G, Barichivich J, Briffa KR, Jones PD. A scPDSI-based global data set of dry and wet spells for 1901–2009. J Geophys Res Atmospheres. 2013;118: 4025–4048. doi:10.1002/jgrd.50355

11. Palmer WC. Meteorological drought. US Department of Commerce, Weather Bureau; 1965.

12. Alley WM. The Palmer drought severity index: limitations and assumptions. J Appl Meteorol Climatol. 1984;23: 1100–1109.

13. Wells N, Goddard S, Hayes MJ. A self-calibrating Palmer drought severity index. J Clim. 2004;17: 2335–2351.

14. Baston, Daniel. exactextractr: Fast Extraction from Raster Datasets using Polygons. 2020. Available: https://CRAN.R-project.org/package=exactextractr

15. Gini index (World Bank estimate) - Latin America & Caribbean, Argentina, Brazil, Chile, Uruguay, Paraguay, Peru, Bolivia, Colombia, Guyana | Data. [cited 17 Jun 2021]. Available: https://data.worldbank.org/indicator/SI.POV.GINI?end=2019&locations=ZJ-AR-BR-CL-UY-PY-PE-BO-CO-GY&start=1979&view=chart

16. Estatística IB de G e. Regiões de influência das cidades 2007. IBGE Rio de Janeiro; 2008.

17. Estatística IB de G e. Regiões de influência das cidades 2018. IBGE Rio de Janeiro; 2020.

18. Wood SN. Generalized additive models: an introduction with R. CRC press; 2017.

19. Fahrmeir L, Kneib T, Lang S. Penalized structured additive regression for space-time data: a Bayesian perspective. Stat Sin. 2004; 731–761.

20. Wood SN. Fast stable restricted maximum likelihood and marginal likelihood estimation of semiparametric generalized linear models. J R Stat Soc Ser B Stat Methodol. 2011;73: 3–36. doi:10.1111/j.1467-9868.2010.00749.x

21. Barcellos C, Lowe R. Expansion of the dengue transmission area in Brazil: the role of climate and cities. Trop Med Int Health. 2014;19: 159–168. doi:10.1111/tmi.12227

22. Brier GW. Verification of forecasts expressed in terms of probability. Mon Weather Rev. 1950;78: 1–3.
