# Supplementary material for: The impact of climate suitability, urbanisation, and connectivity on the expansion of dengue in 21st century Brazil
Source: PLoS Negl Trop Dis. 2021 Dec 9;15(12):e0009773. doi: 10.1371/journal.pntd.0009773 (PMC8691609; doi:10.1371/journal.pntd.0009773)
Supplement: S1 Alternative Language Abstract — (DOCX) [file pntd.0009773.s002.docx]

**S1 Alternative Language Abstract:** Translation of the Abstract into Portuguese by Rafael de Castro Catão.

A Dengue é uma doença hiperendêmica no Brasil, com epidemias ocorrendo em todas as regiões. Estudos anteriores identificaram barreiras geográficas de transmissão no Brasil, em que algumas áreas, como a Amazônia Ocidental e o Sul do Brasil, eram relativamente protegidas de surtos. Dados recentes mostram que as barreiras estão sendo erodidas. Nesse estudo, nos exploramos os fatores de causas principais e identificar os limites atuais da zona de transmissão de dengue. Nós usamos modelo aditivo espaço-temporal para explorar as associações entre as epidemias de dengue e adequação de temperatura, urbanização e conectividade na rede urbana brasileira. O modelo foi aplicado para um indicador binário de epidemias, assumindo o limiar oficial de trezentos casos por cem mil habitantes, para os municípios brasileiros entre 2001 e 2020.  Encontramos uma relação não linear entre altos níveis de conectividade na rede urbana brasileira e a probabilidade de uma epidemia, com menor probabilidade em metrópoles comparada com capitais regionais. O número de meses por ano com condições de temperatura adequadas para mosquitos do gênero Aedes teve associação positiva com a ocorrência de epidemias de dengue.  A adequação de temperatura explicou a maior variação interanual e espacial na Região Sul, confirmando que essa barreira geográfica foi o resultado de temperaturas sazonais mais baixas.  Municípios que já experimentaram epidemias anteriores tinha o dobro da probabilidade de epidemias subsequentes, indicando que a dengue tende a se tornar estabelecidas em áreas após sua introdução. Nós identificamos barreiras geográficas de transmissão a dengue na Região Sul, Amazônia Ocidental e costa norte do Brasil (reentrâncias maranhenses). Embora que a barreira sul ainda exista, ela foi deslocada mais ao sul e a barreira amazônica não possui mais uma fronteira clara. Poucas áreas do Brasil permanecem protegidas de epidemias. Comunidades vivendo nas bordas das antigas barreiras são particularmente susceptíveis para epidemias futuras pela falta de imunidade de grupo. Estratégias de controle deveriam selecionar em áreas de risco para epidemias futuras assim como as que estão atualmente em zonas de transmissão para dengue.
