# Supplementary figures and images for: The impact of climate suitability, urbanisation, and connectivity on the expansion of dengue in 21st century Brazil
Source: PLoS Negl Trop Dis. 2021 Dec 9;15(12):e0009773. doi: 10.1371/journal.pntd.0009773 (PMC8691609; doi:10.1371/journal.pntd.0009773)

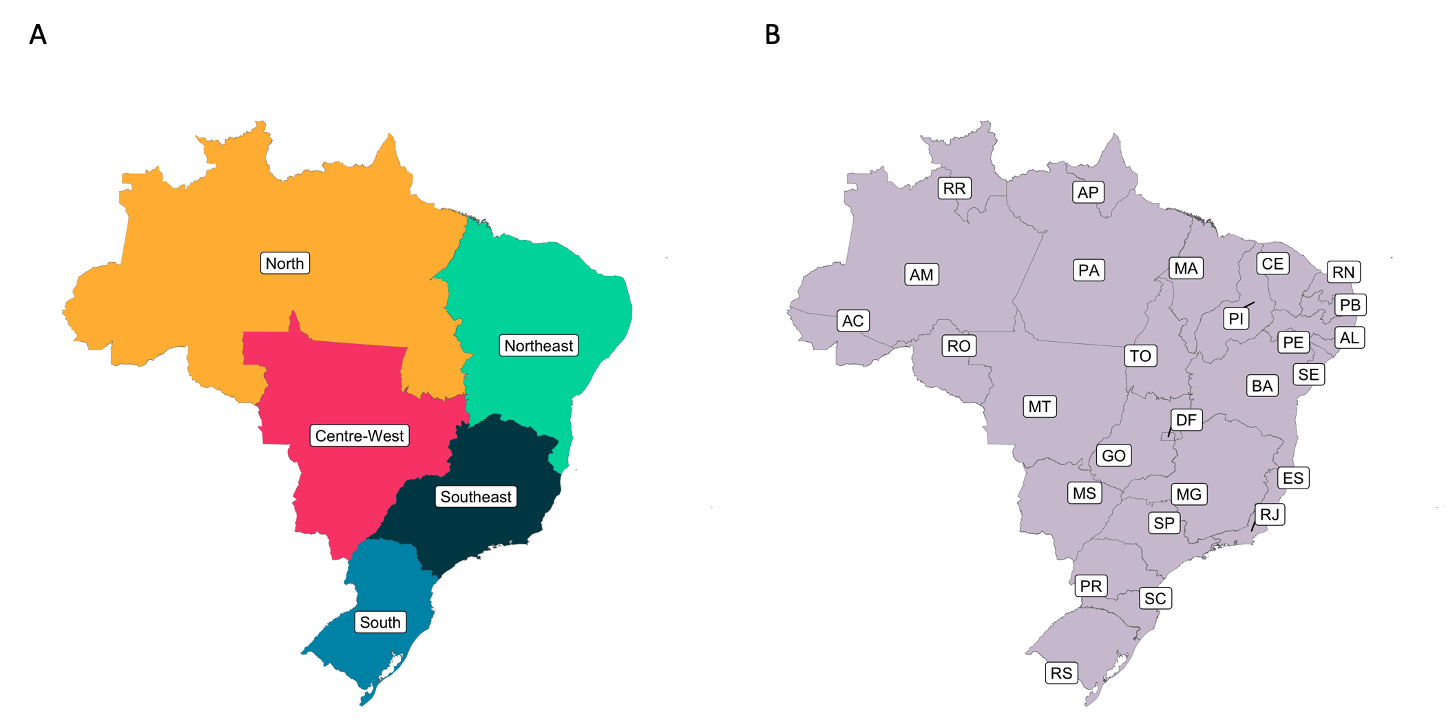

Supplement: S1 Fig — Abbreviations: AC = Acre, AL = Alagoas, AP = Amapá, AM = Amazonas, BA = Bahia, CE = Ceará, DF = Distrito Federal, ES = Espírito Santo, GO = Goiás, MA = Maranhão, MT = Mato Grosso, MS = Mato Grosso do Sul, MG = Minas Gerais, PA = Pará, PB = Paraíba, PR = Paraná, PR = Pernambuco, PI = Piauí, RJ = Rio de Janeiro, RN = Rio Grande do Norte, RS = Rio Grande do Sul, RO = Rondônia, RR = Roraima, SC = Santa Catarina, SP = São Paulo, SE = Sergipe, TO = Tocantins. Maps were produced in R using the geobr package [32,35] (https://ipeagit.github.io/geobr/). (TIF) [file pntd.0009773.s003.tif]

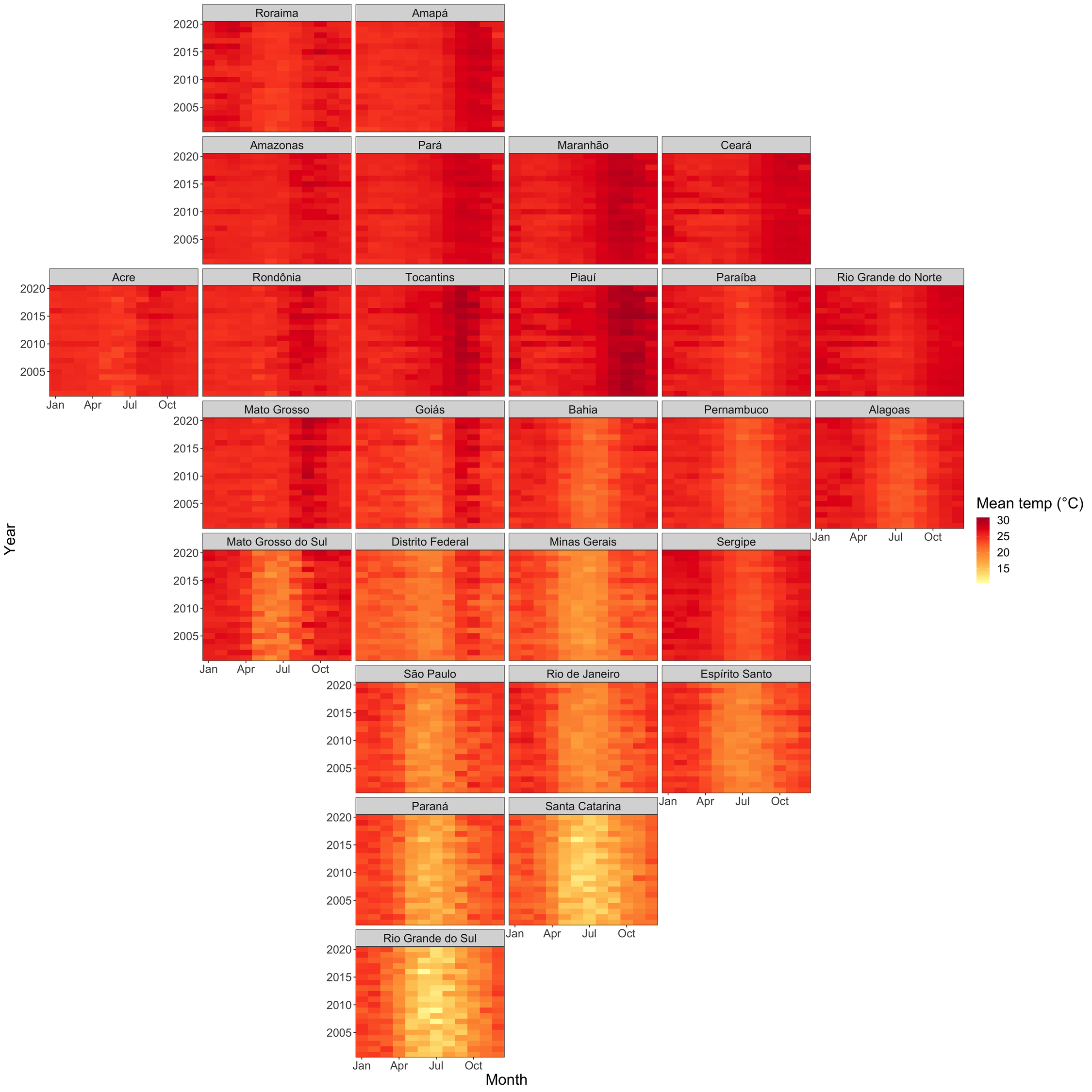

Supplement: S2 Fig — (TIF) [file pntd.0009773.s004.tif]

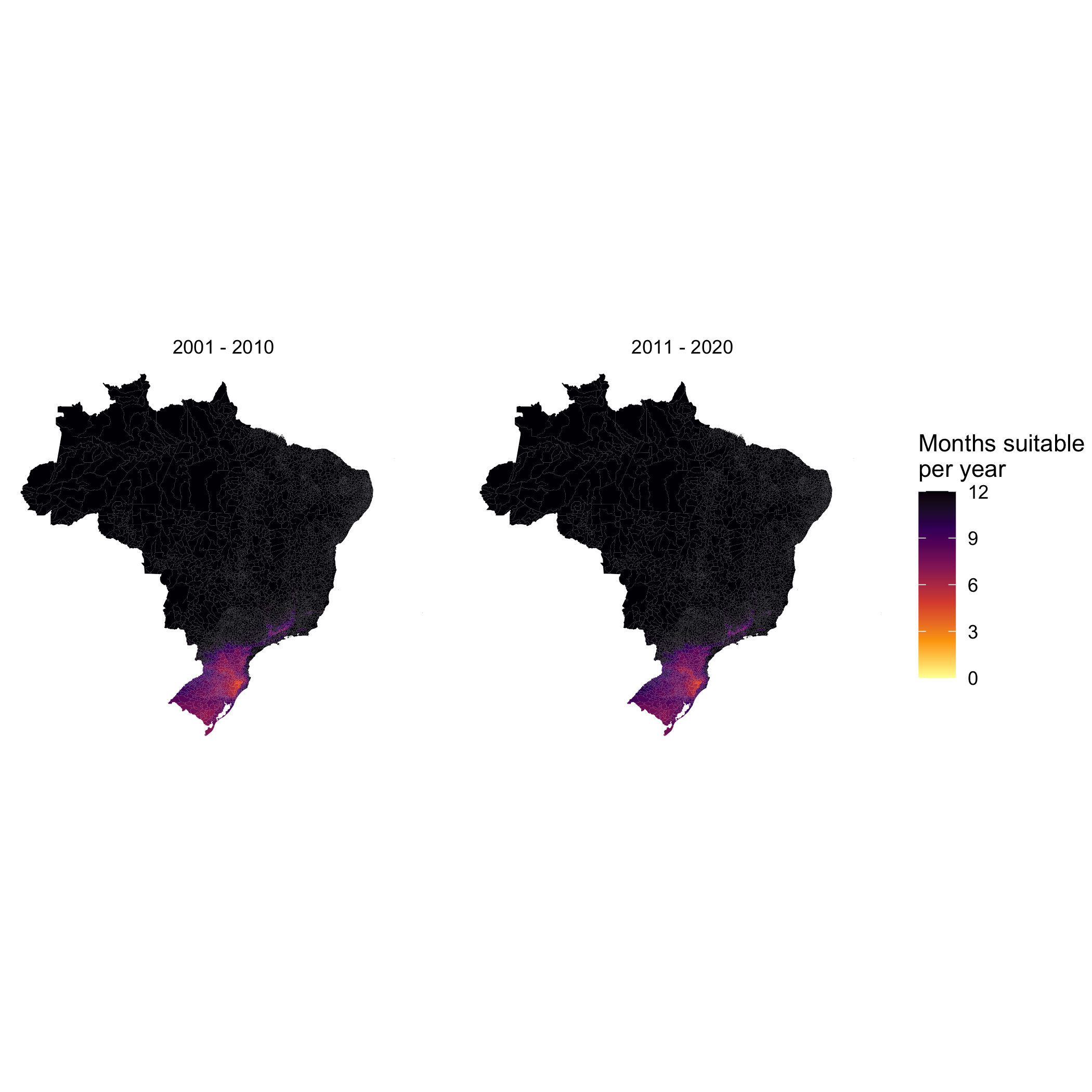

Supplement: S3 Fig — The average number of months with mean temperature between 16.2°C and 34.5°C aggregated to the two decades of data. Most of Brazil experiences suitable temperatures year-round apart from areas of South Brazil and areas of high altitude in the Southeast which experience cool winters. Maps were produced in R using the geobr package [32,35] (https://ipeagit.github.io/geobr/). (TIF) [file pntd.0009773.s005.tif]

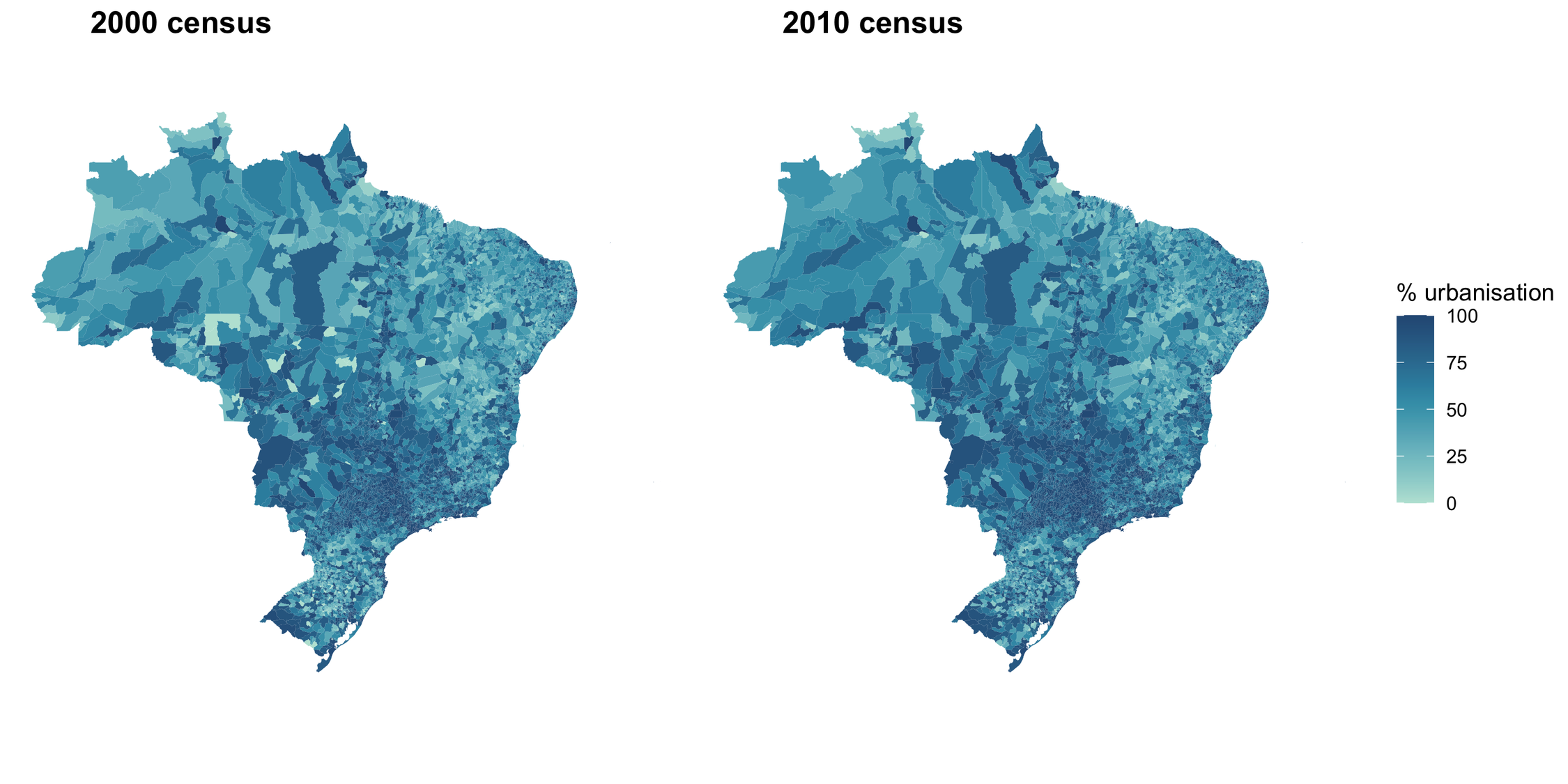

Supplement: S4 Fig — Levels of urbanisation differ greatly across Brazil, with the majority of Southeast and South Brazil living in urban areas in comparison to the North and Northeast which has a larger rural population. Maps were produced in R using the geobr package [32,35] (https://ipeagit.github.io/geobr/). (TIF) [file pntd.0009773.s006.tif]

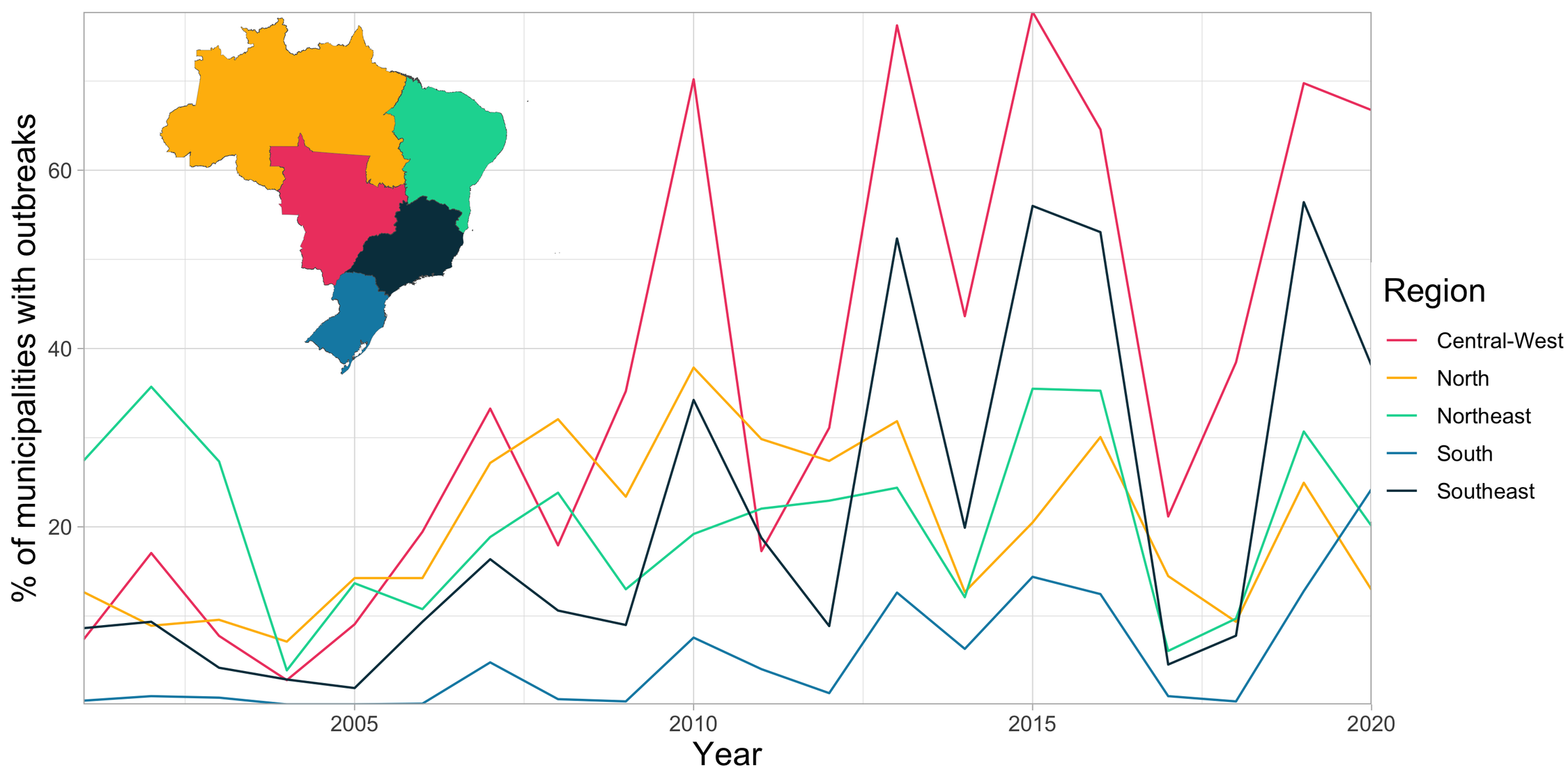

Supplement: S5 Fig — The proportion of municipalities affected by outbreak has increased since 2010 in every region of the country, although outbreaks in South Brazil are still focused on a small part of the region. Maps were produced in R using the geobr package [32,35] (https://ipeagit.github.io/geobr/). (TIF) [file pntd.0009773.s007.tif]

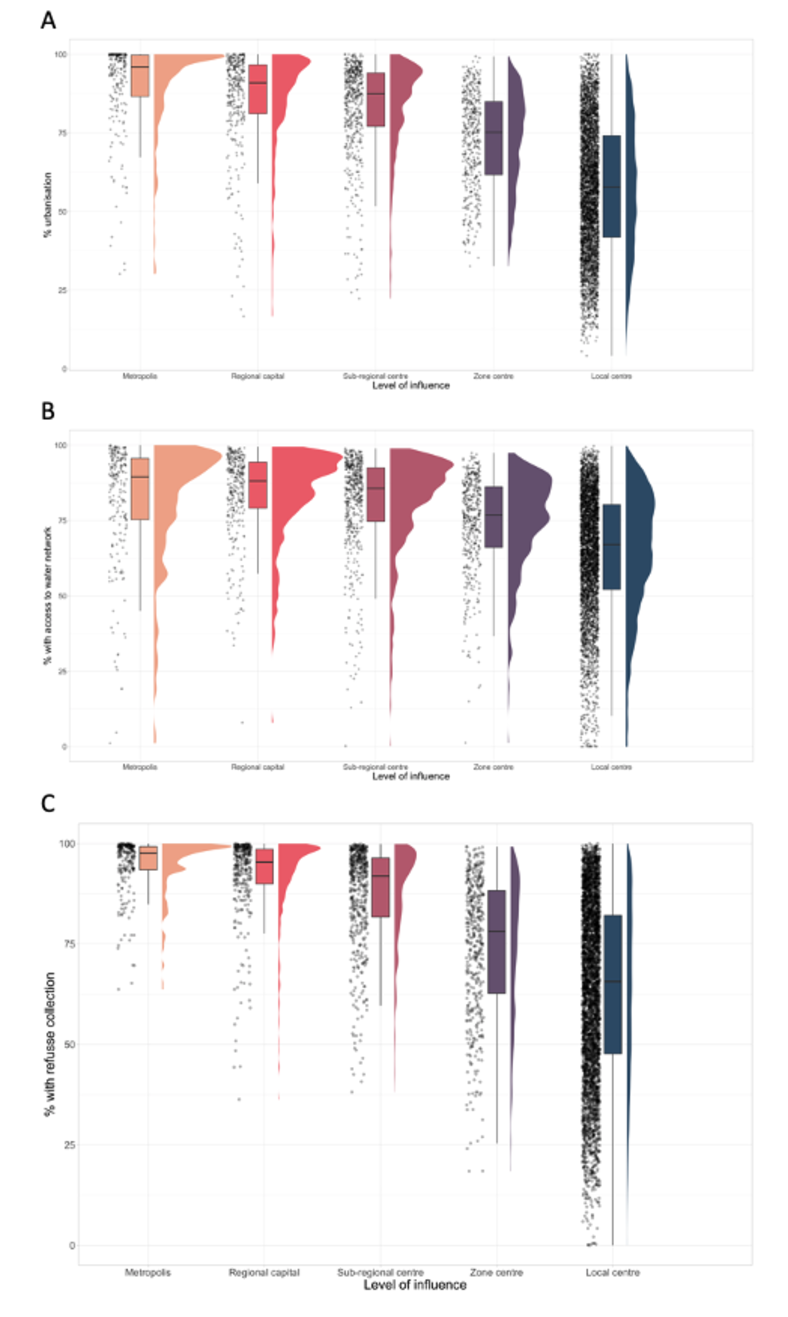

Supplement: S6 Fig — Metropoles and regional capitals have higher levels of urbanisation and access to basic services than municipalities that had lower levels of connectivity within the urban network. Local centres were more varied in terms of basic services and urban levels than the other levels and covered a wide range of city types. (TIF) [file pntd.0009773.s008.tif]

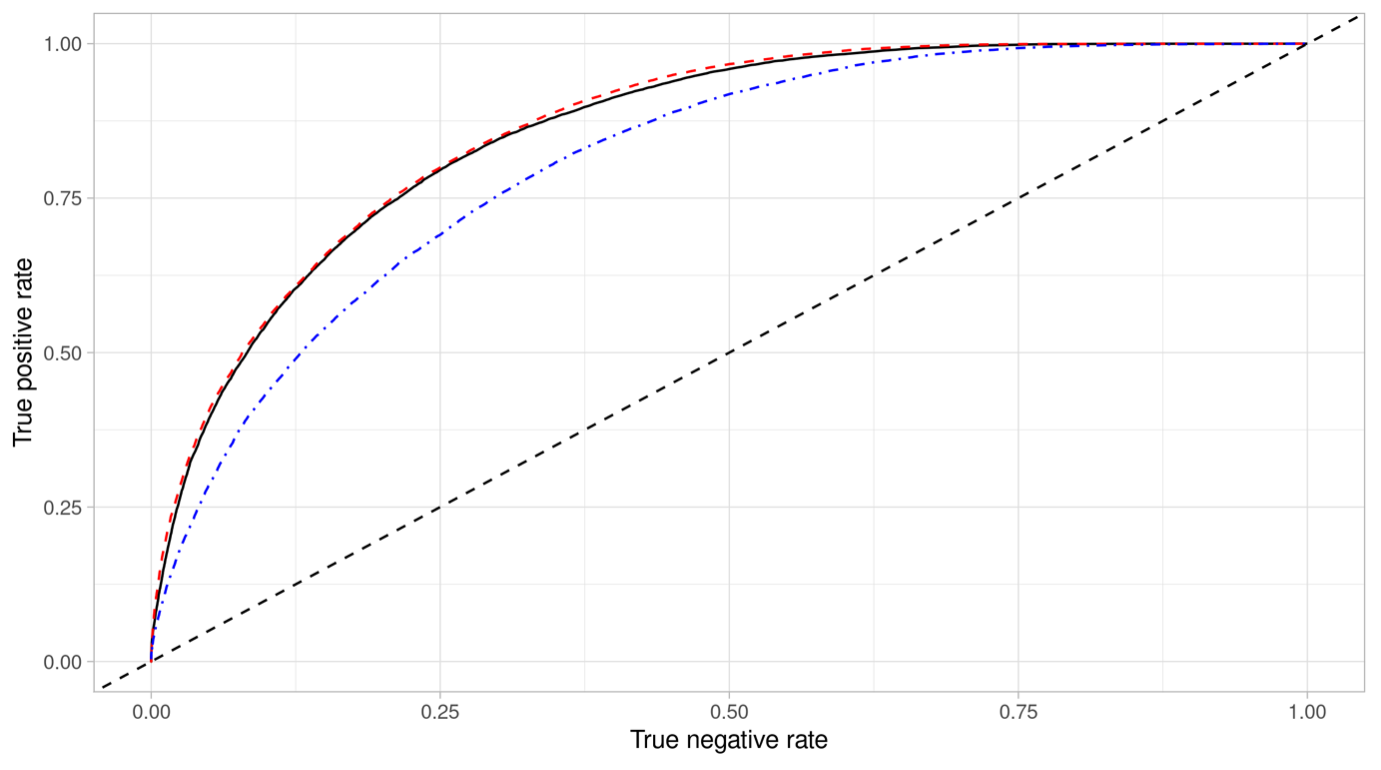

Supplement: S7 Fig — The closer to the top-left corner, the better the predictive ability of a model. As the ROC curve lies above the dashed reference line, this model performs better than chance. (TIF) [file pntd.0009773.s009.tif]

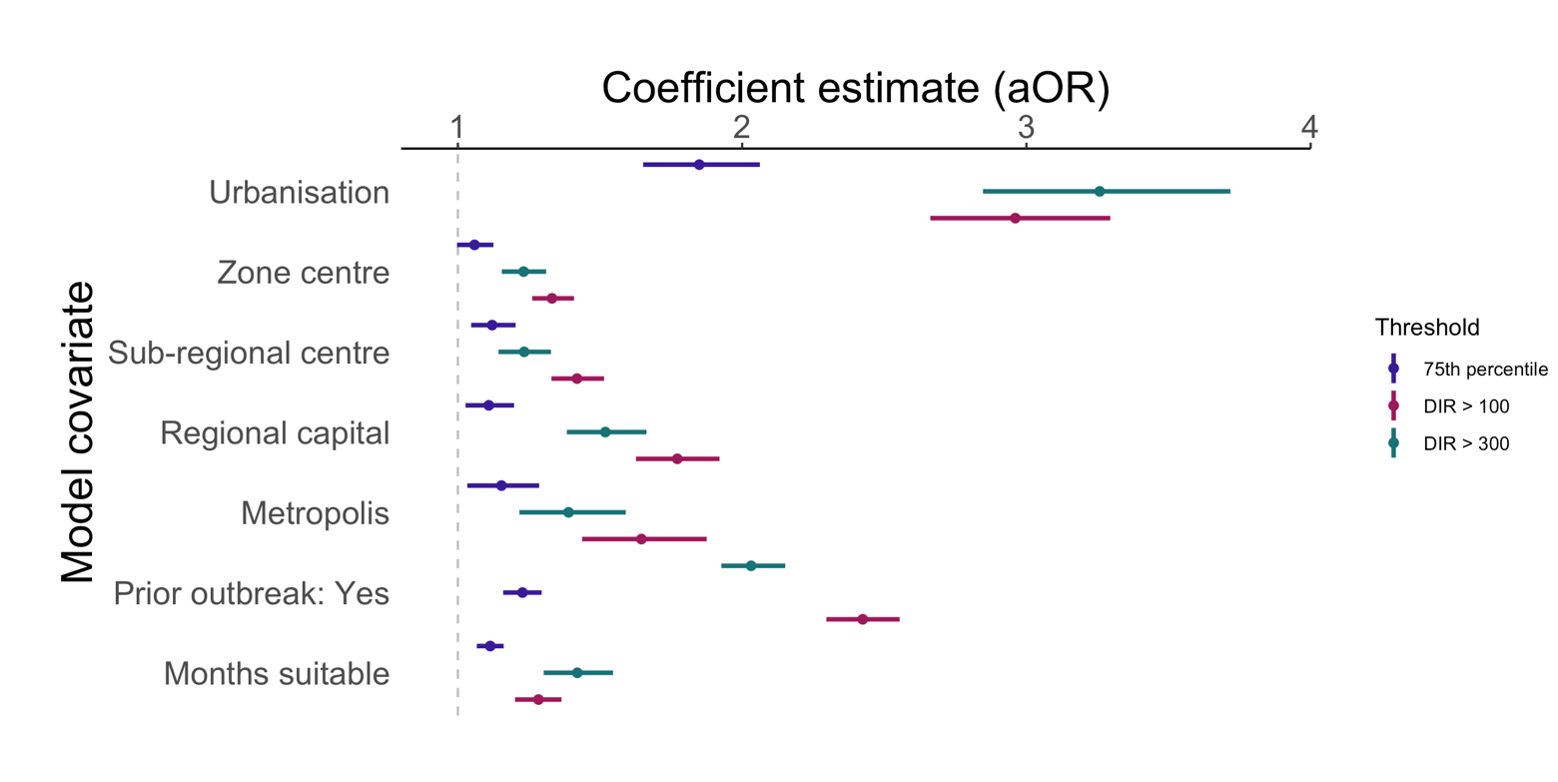

Supplement: S8 Fig — Coefficient estimates using the outbreak indicator based on the 75th percentile were noticeably smaller than the fixed threshold alternatives. The fixed threshold models (where outbreaks were defined as a dengue incidence rate of over 100 or 300) produced similar estimates, however the odds of an outbreak in municipalities after a previous outbreak was higher for the DIR = 100 model. (TIF) [file pntd.0009773.s010.tif]

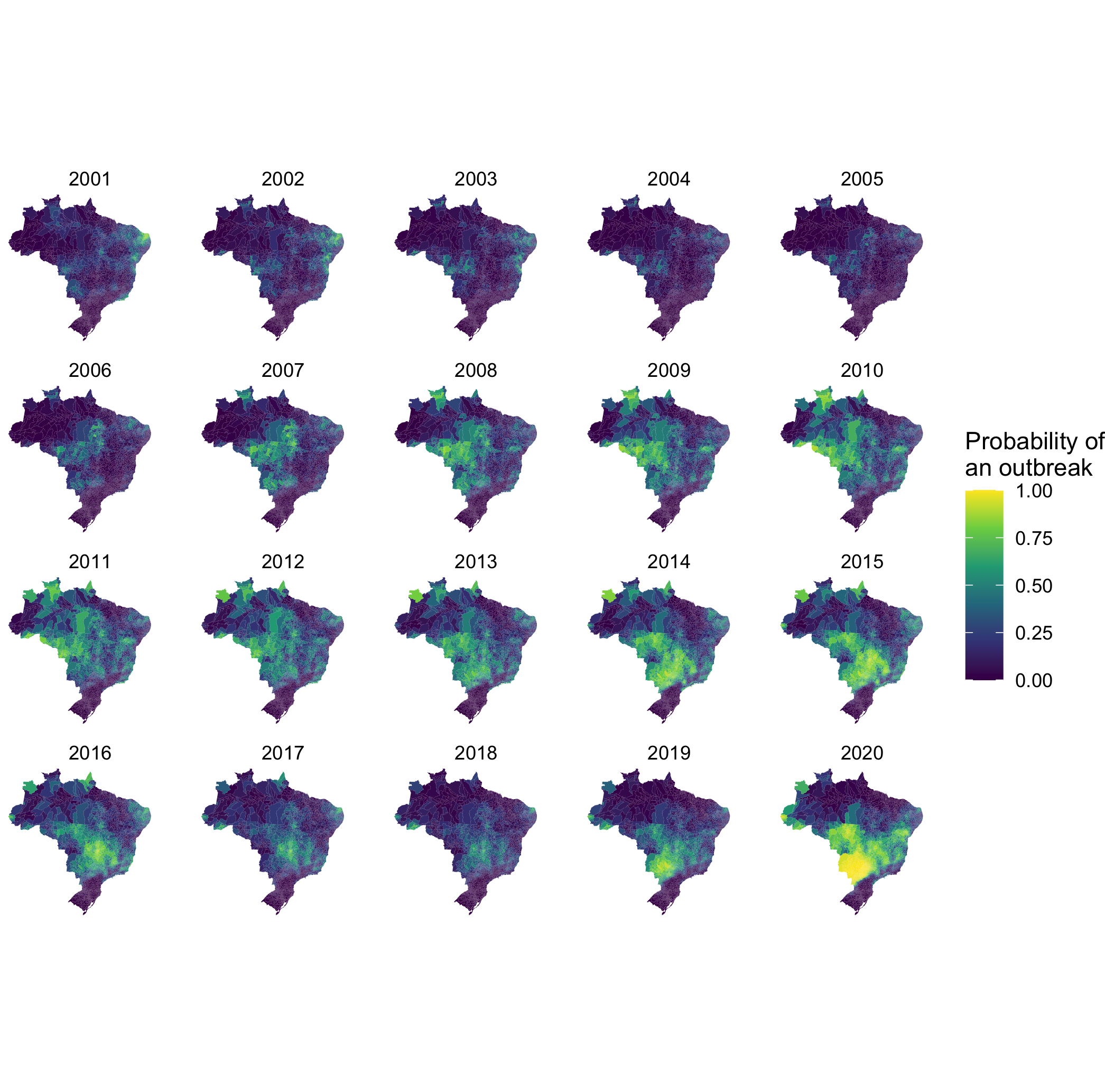

Supplement: S9 Fig — The mean probability of an outbreak estimated by taking 1000 simulations from the posterior distribution of the response and transforming the outcome using a probit function. Maps were produced in R using the geobr package [32,35] (https://ipeagit.github.io/geobr/). (TIF) [file pntd.0009773.s011.tif]

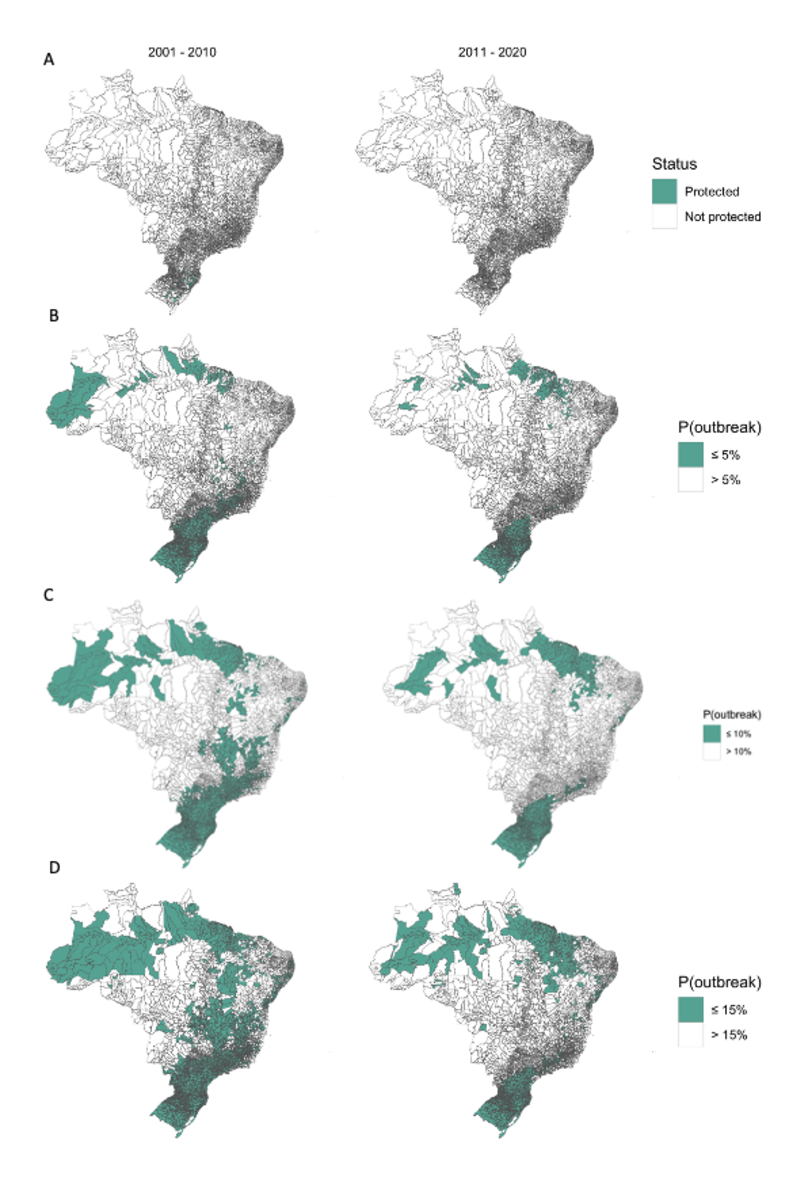

Supplement: S10 Fig — Municipalities were considered ’protected’ if the probability of an outbreak was less than or equal to the threshold a) 0%, b) 5%, c) 10% or d) 15%. The threshold of 10% was chosen as it was the most comparable with previous studies. Maps were produced in R using the geobr package [32,35] (https://ipeagit.github.io/geobr/). (TIF) [file pntd.0009773.s012.tif]
