# Supplementary material for: The impact of climate suitability, urbanisation, and connectivity on the expansion of dengue in 21st century Brazil
Source: PLoS Negl Trop Dis. 2021 Dec 9;15(12):e0009773. doi: 10.1371/journal.pntd.0009773 (PMC8691609; doi:10.1371/journal.pntd.0009773)
Supplement: S2 Table — Coefficient estimates for models assuming an outbreak threshold of over 100 cases per 100,000 (medium risk model), an outbreak threshold of over the 75th percentile of incidence rates, and using temperature suitability for Aedes aegypti only. (DOCX) [file pntd.0009773.s014.docx]

**Table S2: Posterior mean and 95% credible interval (CI) estimates for linear effect parameters, calculated using an outbreak threshold of 100 cases per 100,000 residents, calculated using an outbreak threshold based on the 75th percentile of dengue incidence rate, and using temperature suitability related to *Aedes aegypti* only shown on the adjusted odds ratio (aOR) scale.**

| **Coefficient** | **aOR (95% CI)** | | |
| --- | --- | --- | --- |
|  | **Medium risk model^1^** | **75th percentile model^2^** | **Aedes aegypti model^3^** |
| Urbanisation | 2.96 (2.66, 3.30) | 1.86 (1.66, 2.08) | 3.21 (2.80, 3.65) |
| REGIC level: metropolis | 1.65 (1.44, 1.88) | 1.15 (1.03, 1.28) | 1.38 (1.20, 1.58) |
| REGIC level: regional capital | 1.77 (1.63 1.92) | 1.11 (1.03, 1.21) | 1.51 (1.38, 1.66) |
| REGIC level: sub-regional centre | 1.42 (1.33, 1.51) | 1.12 (1.05, 1.19) | 1.23 (1.14, 1.34) |
| REGIC level: zone centre | 1.33 (1.26, 1.41) | 1.06 (0.99, 1.12) | 1.24 (1.16, 1.31) |
| Prior outbreak: yes | 2.42 (2.30, 2.55) | 1.23 (1.16, 1.29) | 2.01 (1.91, 2.12) |
| Months with suitable temperature | 1.29 (1.20, 1.37) | 1.11 (1.07, 1.16) | 1.34 (1.28, 1.40) |

^1^ Response variable is dengue outbreak defined as over 100 cases per 100,000 inhabitants

^2^ Response variable is dengue outbreak defined as above the 75th percentile of the annual dengue incidence rate between 2001 - 2020, with a minimum incidence equivalent to 5 cases per year.

^3^ Temperature suitability set to *Aedes aegypti* limits, between 17.8° and 34.5°C
