# Supplementary material for: The impact of climate suitability, urbanisation, and connectivity on the expansion of dengue in 21st century Brazil
Source: PLoS Negl Trop Dis. 2021 Dec 9;15(12):e0009773. doi: 10.1371/journal.pntd.0009773 (PMC8691609; doi:10.1371/journal.pntd.0009773)
Supplement: S3 Table — Area under the receiver operator curve and Brier scores for models assuming an outbreak threshold of over 300 cases per 100,000 residents (high risk model), over 100 cases per 100,000 (medium risk model), over the 75th percentile of incidence rates, and a model including the number of months considered extremely wet. (DOCX) [file pntd.0009773.s015.docx]

**Table S3: Model comparison statistics.** Area under the receiver operator curve (AUROC) and Brier scores for models assuming an outbreak threshold of over 300 cases per 100,000 residents (high risk model), over 100 cases per 100,000 (medium risk model), over the 75th percentile of incidence rates, and a model including the number of months considered extremely wet.

| Model formula | AUROC (95% confidence interval) | Brier score |
| --- | --- | --- |
| High risk model | 0.858 (0.856, 0.861) | 0.109 |
| Medium risk model | 0.864 (0.861, 0.866) | 0.138 |
| 75th percentile model | 0.809 (0.807, 0.812) | 0.125 |
| Extremely wet model | 0.859 (0.856, 0.861) | 0.109 |
