# Supplementary material for: The impact of climate suitability, urbanisation, and connectivity on the expansion of dengue in 21st century Brazil
Source: PLoS Negl Trop Dis. 2021 Dec 9;15(12):e0009773. doi: 10.1371/journal.pntd.0009773 (PMC8691609; doi:10.1371/journal.pntd.0009773)
Supplement: Attachment — Submitted filename: Expansion_reviewer_response.docx [file pntd.0009773.s016.docx]

Dear editors and reviewers,

We wish to thank the reviewers for their positive, valuable feedback which we believe has strengthened the analysis and improved the manuscript. We have carefully addressed all the comments (given in italics) as detailed below.

Reviewer #1:

*This study aims at exploring the changes in transmission in Brazilian regions. The goal is to identify the new limits to dengue presence in Brazilian regions and exploring the drivers of dengue expansion using a statistical model. This is a well-written, interesting study with clear assumptions and methodology. I was not familiar with the literature on the association of human transportation networks and dengue spread in Brazil, but the background and approach used were explained very clearly, although I would have liked to understand more what caused differences between urban centers. The analyses and figures are very informative. My only concern is with the choice of threshold as the response variable in the model. I would like to have a better understanding of the choice of incidence chosen, and I would like to see a better comparison between models with different threshold choices. Could we see a sensitivity analysis exploring the impact of 300 cases per 100,000 with*

*other thresholds’ choices (20, 50, etc.) and formal model comparisons? Apart from this I only have a few suggestions.*

We would like to thank reviewer one for their positive, constructive comments. We agree that the choice of a fixed, arbitrary outbreak threshold is not ideal, however these were chosen to reflect the Brazilian Ministry of Health's current definition of 'high risk' (over 300 cases per 100,000 residents) and 'medium risk' (over 100 cases per 100,000 residents) areas (L241 - 243). We chose not to explore other fixed thresholds as we felt they would not be relevant to policy in Brazil and could not find guidance to choose these lower thresholds. We did however include an alternative outbreak definition using the 75th percentile of the dengue incidence rate in each municipality to allow for lower thresholds in municipalities with historically low incidence (further details are given in supplementary material S1). We have also included additional model comparison statistics such as the Brier score and the receiver operator curve for these alternative outbreak definition models based on your feedback (Table S3, Figure S7).

*R1.1: l.126-7. Reference for rank of country by human population size needed.*

A reference has been added to L127.

*R1.2: l.140: I could not find the code associated with this link.*

Thank you for bringing this to our attention, the repository in question has been made public and the code should now be available on GitHub.

*R1.3: l.254-256: Not sure I understand the choice of threshold explored in this analysis. I would appreciate if this could this be specified in the methodology section and in the figure S9 legend. What is the impact of a lower threshold 30, 50 per 100,00?*

The justification for our choice of threshold (previously L142 - 152) has been moved into the modelling approach section (L241 - 243) to make this clearer, we have also added extra analysis and details into the supplementary materials. As explained above, our choice of threshold was based on the Ministry of Health's definition currently used in Brazil: over 300 cases per 100,000 residents is considered 'high risk', and over 100 cases per 100,000 residents is considered 'medium risk'. Although we agree that these arbitrary thresholds are not ideal, they represent the current standard in Brazil which we hope will make our models more relevant to Brazilian policy makers. We did not test alternative fixed thresholds as they do not reflect policy in Brazil and we had no guidance on appropriate values, however we considered alternative thresholds based on percentiles that allowed this threshold to differ across the country. The results of this model are provided in the supplementary material.

*R1.4: l.273: There are many supplementary figures references. It would be helpful to order the plots in the Supplementary material in the order they appear in the text, e.g., here S8 could be ranked S5 and so on.*

Thank you for bringing this to our attention, the supplementary figures are now labelled matching the order they appear in the main text.

*R1.5: l.279: I’m not convinced figure 5 is useful to support this statement. I suggest either removing the sentence and figure or refer to table 1/figure 6 as they present strong evidence of the impact of previous outbreaks.*

We agree this figure did not support the statement in question. We have re-worded this sentence (L297 - 302) to focus on the high incidence rate in the whole region during 2020 (as seen in Figure 3) and the number of municipalities in this state experiencing outbreaks for the first time in 2020 (seen in Figure 4):

"The erosion of the barrier in the South was particularly noticeable in 2020 when it had the highest incidence rate of any region (Figure 3) and many municipalities close to the previous barrier experienced outbreaks for the first time (Figure 4)"

*R1.6: l.306-307: As I said before, my only reserve for this part of the analysis is that it is dependent on the quantitative choice of threshold. I would be interested in seeing a formal quantitative analysis of the relationship between incidence threshold and the likelihood of having more outbreak of similar strength (or at least seeing better support for this choice).*

Although we agree this would be an interesting analysis, it would not be possible to explore the relationship between outbreak thresholds and outbreak strength without using a different response variable (the incidence rate rather than the outbreak indicator). As above, we have moved the justification of our threshold choices into the Modelling approach section to make it clearer and have included another outbreak threshold (the 75th percentile) within the supplementary material.

*R1.7: l.313-314: I’m not sure there is a strong enough evidence of a higher risk of outbreak after an outbreak year. The risk seems to be lower compared to the model with an outbreak any previous year. Could it be outbreaks that overlap over two calendar years instead of a higher risk of outbreak after an outbreak year? A cautious reformulation of this sentence or better explaining the limits of this analysis could help.*

The results given on L313 - 316 were referring to the coefficient values in Table S2 (aOR: 1.18, an 18% increase in the odds of outbreak in municipalities which had an outbreak the previous year, compared to no outbreak in the previous year) rather than a comparison with the 'any prior outbreak' model. However, we agree that an outbreak in the previous year may not reflect outbreaks spanning multiple years, therefore we have removed this indicator from the model.

*R1.8: l. 340-349: Could it be that other confounders exists that may affect dengue reporting rates? E.g., regional centers having a higher number of doctors per capita than metropoles?*

This is an interesting consideration, however the number and type of medical institutions is one of the factors that was used to categorise cities into the levels of influence so the metropoles have more doctors and health facilities than regional capitals. We believe that the issue may be the inequality of access to these services within larger cities but unfortunately, we have not been able to source reliable data that accounts for this at the municipality level. We have amended the interpretation of these. results on L363 - 365 to the following:

"metropoles have larger economies than regional capitals and greater access to healthcare (29) which may mean improved infrastructure which is not reflected by census variables at this scale."

*R1.9: l.351-352. I agree that the models reach to the same conclusions, but I do see differences in the parameter estimates where the estimates for some models were not within the bounds of the main model. Also, I am curious what the ROC figure (S9) looks like for the other model formulations (100 per 100,000 incidence, model with each covariate), could they be plotted in dashed or grey-ish line?*

We have extended our comparison of models with different outbreak thresholds (including the 75th percentile model) into the supplementary material. We agree that these coefficient values were different so to highlight this, we have added Figure S8 which shows the coefficient estimates with 95% credible intervals on the same plot and have added the following to L368-373:

"We found the conclusions drawn from the models using alternative outbreak definitions remained consistent, however the coefficient values differed (Figure S8, Table S2). In particular, the model based on the 75th percentile produced lower coefficient estimates than the fixed threshold models, and the model using a threshold of over 100 cases per 100,000 residents estimated an increased odds following a previous outbreak compared to the primary analysis (Figure S8, Table S2)."

We have also included the ROC curve for the alternative threshold models into Figure S7.

*R1.10: l. 354-358: Very informative part of the analysis! I really appreciate the information and the visualization of the spatially heterogeneous risk of dengue outbreak.*

Thank you!

*R1.11: l. 375: In some instances, “climate suitability” is used but temperature suitability may be more appropriate as the temperature is the only variable used for this parameter estimate (and the words "temperature suitability" are used in the methods section).*

Thank you for bringing this to our attention, we have checked the paper to ensure that the language is consistent when referring to temperature suitability.

*R1.12: l.407-409: Methods for this analysis? Since the figures for this analysis are presented in the main text the methods, could be found in the methods section of the main text?*

Thank you for this observation, the methods have been moved to the Methods section (see L278-283 in the main text)

Reviewer #2:

*R2.1: Study by Lee et al. built upon previous literature that showed geographic expansion of dengue transmission in Brazil, and it updated the changing geographic barrier to dengue transmission in Brazil. It explored several drivers behind such expansion and provided additional evidence on the impact of temperature suitability and urbanization on dengue outbreaks in Brazil. The manuscript is well-written and has important policy implications.*

We would like to thank reviewer 2 for their positive comments and for recognising the importance of our findings on policy. We have addressed your concerns below:

***Methods***

*R2.2: The authors only explored one environmental factor, which is temperature suitability, for explaining the expansion of dengue. Other climatic factors such as rainfall and humidity may also impact the Aedes aegypti vector population. Please explain the rationale behind choosing temperature as the only environmental factor here.*

We agree that other climatic factors such as precipitation and humidity influence vector populations and therefore transmission dynamics, however we chose to primarily focus on temperature as previous studies had shown that this was a main contributor to the dengue expansion barriers that we wished to explore. Unlike temperature, we could not find clear agreement in the literature about suitable limits of precipitation and humidity for Aedes mosquitoes to transmit dengue. Although we considered exploring the impact of changes in humidity levels and precipitation on dengue expansion, the differences in climate systems across Brazil means these anomalies would likely have different impacts in different regions. To explore the impact of hydrometeorological factors on dengue, we have included the number of months considered 'extremely wet', defined using the self-calibrating Palmer Drought Severity Index (scPDSI), as a covariate (see the supplementary material for more details). Although the result agreed with previous literature that transmission increases following extremely wet conditions, we found the inclusion of wet months did not improve the model so did not include it in our primary analysis.

*R2.3: The study created a binary outbreak indicator based on case counts in the dengue surveillance system in Brazil as the response variable of the GAM model. Please comment on how the surveillance system is sensitive to change in health seeking behavior or access to healthcare over time, and how it may impact the model output.*

We had previously included discussion of how differences in the surveillance system could introduce bias into the analysis in the supplementary material, however based on your comments we have added this discussion to the main text (L135 - 140). We agree that the surveillance system is sensitive to changes in health-seeking behaviour (this is particularly notable between epidemic and non-epidemic periods), however by using outbreak indicators as our response variable we hope to reduce this bias. Although the incidence rates themselves may not be accurate, they will be able to detect whether an outbreak has occurred or not.

***Results***

*R2.3: The results are clear overall. One minor comment is that under "model result" on line 309, the authors stated that the increased OR suggests that dengue becomes established once the virus is introduced. It would be helpful to present dynamics of the four circulating serotypes otherwise the statement may be a stretch.*

We agree that this interpretation requires more biological evidence, unfortunately we have very little information about the serotypes from the surveillance system (between 2014 - 2020, less than 0.5% of the cases in our data had information about serotype). We have amended this interpretation on L330 - 333 to the following:

"This could indicate that the virus becomes established following its introduction, however the increased incidence may be a result of increased surveillance following an outbreak or due to the increased probability of severe cases following the introduction of new serotypes"

***Conclusion***

*R2.4: The conclusions are clear and well presented.*

Thank you!
